# AMP-Activated Protein Kinase Activation during Cardioplegia-Induced Hypoxia/Reoxygenation Injury Attenuates Cardiomyocytic Apoptosis via Reduction of Endoplasmic Reticulum Stress

**DOI:** 10.1155/2010/130636

**Published:** 2011-01-23

**Authors:** Chi-Hsiao Yeh, Tzu-Ping Chen, Yao-Chang Wang, Yu-Min Lin, Shu-Wen Fang

**Affiliations:** ^1^Department of Thoracic and Cardiovascular Surgery, Chang Gung Memorial Hospital, 222 Mai-Chin Road, Keelung 204, Taiwan; ^2^College of Medicine, Chang Gung University, 259 Wen-Hwa 1st Road, Tao-Yuan 333, Taiwan

## Abstract

Cardioplegic-induced H/R injury results in cardiomyocytic apoptosis. AMPK has been shown to reduce ER stress and the unfolded protein response (UPR). Whether AMPK activation can attenuate cardiomyocytic apoptosis after cardioplegia-induced H/R injury is unknown. Cardiomyocytes were exposed to simulated ischemia by incubation in a hypoxic chamber with intermittent cold cardioplegia solution infusion at 20-minute intervals and subsequently reoxygenated in a normoxic environment. Various doses of AMPK activators (AICAR or metformin) were given 2 days before H/R injury. The cardiomyocytes were harvested after reoxygenation for subsequent examination. With both AMPK activators, the antiapoptotic genes of ER stress and UPR, the subsequent production of proapoptotic proteins was attenuated, and the antiapoptotic proteins were elevated. The activity of the apoptotic effectors of ER stress was also reduced with AMPK activation. Moreover, TUNEL staining showed that AMPK activation significantly reduced the percentage of apoptotic cardiomyocytes after cardioplegia-induced H/R injury. Our results revealed that AMPK activation during cardioplegia-induced H/R injury attenuates cardiomyocytic apoptosis, via enhancement of antiapoptotic and reduction of proapoptotic responses, resulting from lessening ER stress and the UPR. AMPK activation may serve as a future pharmacological target to reduce H/R injury in the clinical setting.

## 1. Introduction

AMPK is an enzyme that is activated when cellular energy is depleted [1, 2]. AMPK activation increases glucose uptake and fatty acid oxidation in skeletal muscle cells to increase ATP production [3–6]. The AMPK cascade has come to prominence as a sensor of metabolic stress that appears to be ubiquitous throughout eukaryotes [7, 8]. AMPK is activated by many different metabolic stresses, including heat shock and metabolic poisons in hepatocytes [9], exercise in skeletal muscle [10], and ischemia and hypoxia in the heart [11]. We previously proposed that the activation of the mitochondria-mediated apoptotic pathway has a major role in the development of cardiomyocytic injury and myocardial dysfunction during cardioplegia-induced cardiac arrest under cardiopulmonary bypass [12–14]. The association between the pathogenesis of cardioplegia-induced hypoxia/reoxygenation (H/R) injury and its complications and endoplasmic reticulum (ER) stress has not been fully elucidated. AMPK activation has been shown to reduce ER stress and the unfolded protein response (UPR). Because 5-aminoimidazole-4-carboxamide ribonucleoside (AICAR) has been reported to exert a possible additional benefit in preventing ER stress and the UPR [15], we investigated the effects of AICAR on ER stress and the UPR in cultured HL-1 cardiomyocytes. 

Studies using cultured cardiomyocytes have helped elucidate the mechanisms involved in the response to H/R injury induced by cardioplegia, which induces cardiomyocytic damage through a variety of mechanisms, including cardiomyocytic apoptosis [12]. Our previous studies revealed that cardioplegia-induced H/R injury could result in cardiomyocytic injury, characterized by markers of apoptosis, including cytochrome *c* release, caspase activation, and DNA fragmentation. Although relatively few studies have focused on mechanisms of H/R-induced cell death in cardiomyocytes, recent evidence obtained using cultured cardiomyocytes suggested that cardioplegia-induced H/R injury resulting cardiomyocytic apoptosis might be initiated via several mechanisms, including decreased synthesis of the signature mitochondrial membrane phospholipid, cardiolipin [16] and increased generation of reactive oxygen species [17, 18]. However, the induction of apoptosis by oxidative stress requires a flux of calcium ions from the ER to the mitochondria, and depletion of these calcium stores can impair normal protein-folding functions, leading to ER stress [17, 18]. Consistent with this concept, our present study was designed to show whether cardioplegia induced H/R insult could induce ER stress in cardiomyocytes. Furthermore, our studies would clarify if ER stress after cardioplegia-induced H/R injury could be attenuated, in part, through the activation of AMPK pathway by AICAR or metformin.

## 2. Material and Methods

The HL-1 cell line [19], obtained from Claycomb Lab (Louisiana State University Medical Center, NO), was used as the experimental model. HL-1 is an immortal cell line with a phenotype that is similar to adult cardiomyocytes. The characteristics of HL-1 cells include the following: (a) an ultrastructure similar to primary cultures of adult atrial cardiac myocytes; (b) cytoplasmic reorganization and myofibrillogenesis; (c) the presence of highly ordered myofibrils and cardiac-specific junctions; (d) the ability to undergo spontaneous contractions; (e) presence of several voltage-dependent cardiac-specific currents. These properties make HL-1 cells particularly attractive for studies that require expression of adult-specific genes and for studies of cardiomyocyte function [19].

## 3. Perfusion System

The perfusion system was set-up as Vanden Hoek et al. previously described [20]. In brief, coverslips with synchronously contracting cells were placed inside a Sykes-Moore chamber (1.2 mL; Bellco Glass Inc., Vineland, NJ, USA). The chamber and inflow tubing were water jacketed, and oxygen leaks were minimized. The temperature, flow rate, pH, and PO_2_ inside the chamber were continuously monitored and held constant. Hypoxia within the chamber was verified by perfusing the system with zinc-free methylene blue chloride, which changes from clear to vivid blue at a PO_2 _> 1.5 Torr. The standard perfusion medium consisted of Claycomb medium.

## 4. Groups

Group 1: HL-1 cardiomyocytes were perfused with warm (37°C) oxygenated culture medium.
Group 2H: HL-1 cardiomyocytes were perfused with deoxygenated hypothermic (4°C) cardioplegia every 20 minutes for 120 minutes.
Group 2R: HL-1 cardiomyocytes were perfused with deoxygenated hypothermic (4°C) cardioplegia every 20 minutes for 120 minutes then reperfused with warm (37°C) oxygenated culture medium for 16 hours.
Groups 3–6: 0.01, 0.1, 0.5, and 2 mmol/L metformin were, respectively, added to the culture medium for 2 days then subjected to the same protocol as Group 2R.
Groups 7–10: 0.01, 0.1, 0.5, and 2 mmol/L AICAR were, respectively, added to the culture medium for 2 days then subjected to the same protocol as Group 2R.

## 5. Western Blot Analysis

Western blot analysis was performed on HL-1 cardiomyocytes from the different groups, as previously described [14]. The cells were lysed and centrifuged, then the supernatants were used as sample proteins. Samples were denatured and then separated through an SDS-polyacrylamide gel and transferred to polyvinylidene difluoride membranes. The membranes were rinsed, then blocked for 1 h, and incubated overnight at 4°C with either anti-AMPK*α*_1_ antibody, anti-AMPK*α*_2_ antibody (Upstate, Lake Placid, NY), or anti-AMPK phosphothreonine 172-specific antibody (Cell Signaling Technology, Beverly, MA). Immunodetection was accomplished by incubating the membranes with a goat antirabbit IgG (Santa Cruz Biotechnology, Santa Cruz, CA) in TBS-Tween for 1 h. Visualization was performed with a BM Chemiluminescence Blotting Substrate kit (Roche Diagnostics, Mannheim, Germany), according to the instructions provided by the manufacturer. The immunoreactive bands were quantified by digital densitometric imaging (Gel Doc 1000 with a Model GS-700 Densitometer and Molecular Analyst Software, BioRad). Cell levels of phosphorylated eIF-2*α* (Cell Signaling Technology, Beverly, MA) and GADD153 (Santa Cruz, CA), Grp78, Bcl-2/Bax ratio, Caspase-3, -12, and cleaved PARP (PharMingen, San Diego, CA) protein were also determined by western blot analysis as described above. Signal intensities of specific product bands in the western blot were normalized with those of *β*-actin or tubulin to correct for differences in manipulation and expressed as an increase (fold) of protein expression compared with those in Group 1.

### 5.1. Caspase-3 and AMPK Activities

Caspase-3 activity was determined by measuring proteolytic cleavage of the specific substrate T*n*T-acetyl-Asp-Glu-Val-Asp-7-amino-4-trifluoromethyl coumarin (Ac-DEVD-AFC; Biomol, Plymouth Meeting, PA) in the presence or absence of the specific inhibitor T*n*T-acetyl-Asp-Glu-Val-Asp-CHO (Ac-DEVD-CHO; Biomol) as previously described [14]. AMPK activity was measured using homogenizing HL-1 cardiomyocytes in accordance with the manufacture's protocol (CycLex Co., Ltd, Nagano, Japan). Briefly, the cardiomyocytic lysates from various groups were added on a precoated plate with a substrate peptide corresponding to mouse insulin receptor substrate-1. The phosphorylation of insulin receptor substrate-1 was detected with an antimouse monoclonal antibody and peroxidase-coupled antimouse IgG antibody, which could be quantitated by absorbance measurement. The activities of each group were expressed as an increase (fold) of activities compared with those in Group 1.

## 6. Quantitative Real-Time PCR and Analysis of ATF 6, VEGF, Grp78, and Spliced XBP-1

Quantitative real-time PCR reactions were performed on a Roche LightCycler Instrument 2.0 with LightCycler TaqMan Master (Roche Applied Science, Indianapolis, IN). Briefly, 20 mL of reaction solutions contained 5 mL generated cDNA template, 4 mL Master Mix, 0.2 mL of 10 mM probe, 0.4 mL of 10 mM forward primer, 0.4 mL of 10 mM reverse primer, and 10 mL water. The q-RT PCR program was conducted at 95°C for 10 min, 45 cycles of 95°C for 10 s, 72°C for 1 s, and 40°C for 30 s. At the end of the programs, melt curve analysis was performed. At the end of each q-RT-PCR run, the data were automatically analyzed, and an amplification plot was generated for each cDNA sample. From each of these plots, the LightCycler4 Data analysis software automatically calculated the crossing point (CP) value, which indicates the beginning of exponential amplification. The mRNA level was normalized with reference to the amount of the housekeeping gene transcript (glyceraldehyde-3-phosphate dehydrogenase (GAPDH) mRNA). Reverse transcription PCR was performed as previously reported [12–14] primers. The primer sequences for spliced XBP-1, with a 229-bp product, were as follows: forward primer 5′-GGCCTTGTGGTTGAGAACCAGGAG-3′ and reverse primer 5′-GAATGCCCAAAAGGATATCAGACTC-3′. The signal intensities of specific PCR product bands were normalized with those of the GAPDH band to correct for differences in manipulation and expressed as an increase (fold) of mRNA expression compared with those in Group 1.

## 7. Detection of Apoptotic Cardiomyocytes 

In brief, cells were fixed to slides and rinsed with terminal deoxynucleotidyl transferase (TdT) buffer. Then the slides were incubated with TdT (2.5 *μ*L), biotin-dUTP (2.5 *μ*L), and TdT buffer (45 *μ*L) in a moist chamber at 37°C for 60 minutes. The reaction was terminated by transferring the slides to a buffer containing 300 mmol/L sodium chloride and 30 mmol/L sodium citrate for 15 minutes at room temperature. Cardiomyocytes were stained with terminal deoxynucleotidyl transferase dUTP nick end labeling (TUNEL) reagent and anti-Myc antibody followed by antimouse IgG rhodamine. After coverslip mounting with 90% glycerol and 10% phosphate-buffered saline, the slides were examined by fluoromicroscopy. Apoptotic nuclei containing nicked DNA stained green and were present in all of the groups.

## 8. Data Analysis

Statistical comparison of the differences among groups was performed by analysis of variance for normally distributed data or by the Kruskal-Wallis test for data that did not follow a normal distribution. When the ANOVA or Kruskal-Wallis tests indicated that a significant difference was present, a Student's *t*-test or Mann-Whitney test was used, respectively, to compare differences between pairs of groups. Statistical significance was assumed when the *P* value was <.05.

## 9. Results

### 9.1. Effect of AMPK Activation on AMPK Production during Cardioplegia-Induced H/R Injury

**Figure 1:**
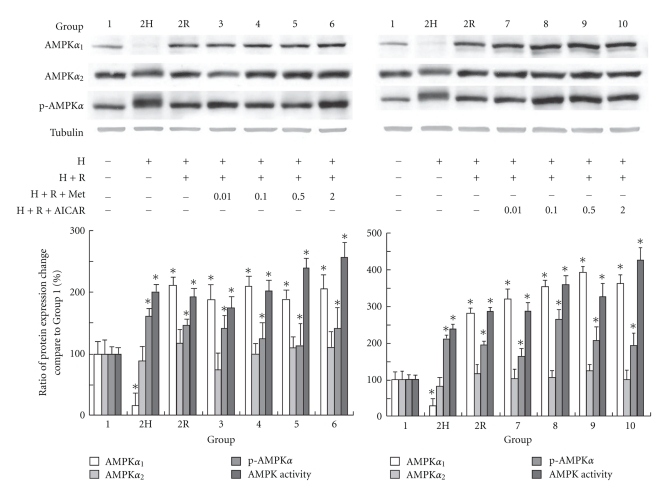
The results of western blot analysis of AMPK*α*_2_, AMPK*α*_2_, and p-AMPK*α* and AMPK activities in different groups are shown. Tubulin was used as internal control. (Results are representative of at least 3 separate experiments. *P < .05 compared with Group 1; H: hypoxia; R: reoxygenation; Met: metformin.)

Western blot analyses of proteins from the cardiomyocytes confirmed the presence and differential expression of the AMPK in the various groups (Figure 1). To ameliorate any confounding influences on protein expression, we normalized the expression of AMPK to tubulin in each group, respectively. A significant increase of AMPK*α*_1_ expression compared with that in Group 1 was observed in all groups except for the hypoxia-only group (Group 2H) that showed a significant decrease of expression. The expression of AMPK*α*_2_ among all groups was similar to the expression level in Group 1. However, the elevation of phosphorylated AMPK in all groups was significantly different from that in Group 1. These results suggest that hypoxia, H/R, or AMPK activation with metformin or AICAR increases AMPK phosphorylation, which could be used to estimate the kinase activity of *α*_2_-AMPK and *α*_1_-AMPK based on AMPK Thr^172^ phosphorylation. The AMPK activities of Groups 3–6 were significantly elevated compared with Group 1. The AMPK activities elevated even more with higher concentration of metformin administration. The similar effect could be seen in the AICAR administration groups.

### 9.2. Effect of AMPK Activation on Expression of EIF-2*α* Phosphorylation and Downstream Proteins in HL-1 Cardiomyocytes

**Figure 2:**
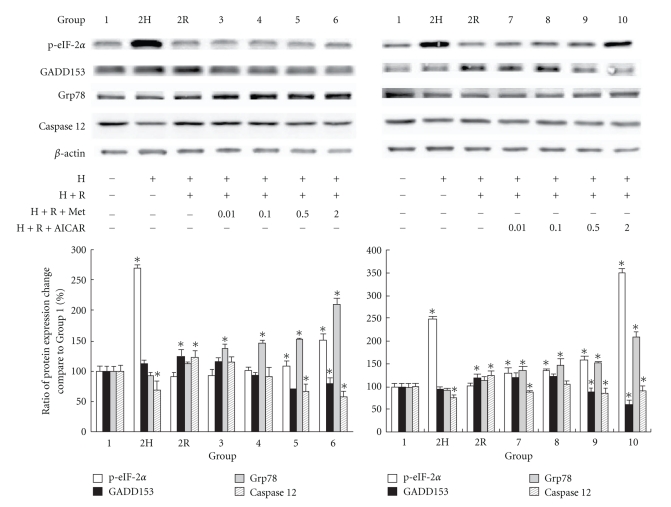
The results of western blot analysis of phosphorylated eIF-2*α*, GADD153, Grp78, and caspase-12 (B) in different groups are shown. *β*-actin was used as internal control. (Results are representative of at least 3 separate experiments. *P < .05 compared with Group 1; H: hypoxia; R: reoxygenation; Met: metformin.)

Eucaryotic translation initiation factor 2 (eIF-2*α*) kinase is one of the proteins that is phosphorylated in the initial response of the cell to ER stress, and this activation attenuates translation initiation and protein synthesis to reduce ER stress and subsequent UPRs [21]. After 2 hours of hypoxia, the eIF-2*α* phosphorylation was significantly elevated, as shown in Figure 2. However, the elevation of phosphorylated eIF-2*α* diminished when hypoxia was followed by 16 hours of reoxygenation, except in the groups with a higher concentration of metformin and AICAR. The expression of phosphorylated eIF-2*α* in Groups 5, 6, 9, and 10 was significantly higher than that in Groups 1 and 2R. The UPR induces the expression of 78-kDa glucose-regulated protein (Grp78), an ER-resident molecular chaperone that prevents the aggregation of unfolded or misfolded proteins so that they can be properly refolded [22]. The expression of Grp78 can be induced by three branches of the UPR, including eIF-2*α* phosphorylation branch. Grp78 may form a complex with caspase-7 and -12 at the ER surface, preventing their activation and release. As shown in Figure 2, the protein levels that followed eIF-2*α* phosphorylation, such as Grp78, GADD153, and caspase-12 (the downstream executor of ER stress) were evaluated with western blot analysis. The levels of antiapoptotic Grp78 protein were significantly elevated in Groups 3–10 compared with that in Group 1 (Figure 2), indicating the activation of AMPK with either metformin or AICAR. In contrast, the expression of proapoptotic protein GADD153, which was significantly elevated after reoxygenation (Group 2R versus Group 1, *P* < .05), was significantly decreased with AMPK activation. The subsequent activation of caspase-12, which is controlled by the balance between Grp78 and GADD153 expression, was elevated after H/R injury. However, the elevation was diminished with either of the two AMPK activators that we tested.

### 9.3. Effects of AMPK Activation on UPR Target Gene Expression in HL-1 Cardiomyocytes during Cardioplegia-Induced H/R

**Figure 3:**
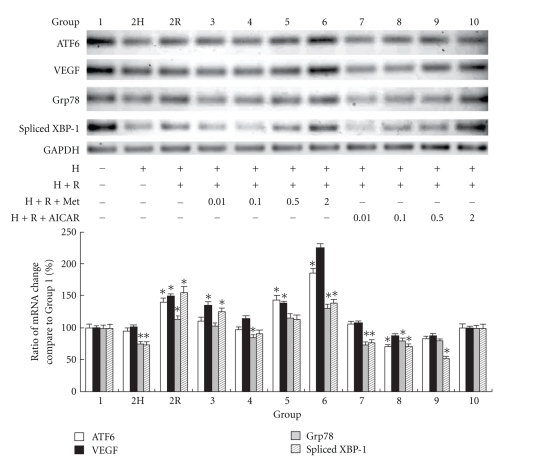
Reverse transcription PCR analysis showed up-regulation of ATF6, VEGF, Grp78, and spliced XBP-1 mRNAs in HL-1 cardiomyocytes from different groups. Glyceraldehyde-3-phosphate dehydrogenase (GAPDH) served as the loading control. The PCR products of GAPDH, ATF6, VEGF, Grp78, and spliced XBP-1 were designed to be around 200 bp. Quantitative real-time PCR showed an elevated ratio of these mRNAs in various groups. (Results are representative of at least 3 separate experiments. *P < .05 compared with Group 1; H: hypoxia; R: reoxygenation; Met: metformin.)

Figure 3 shows the expression of these transcripts using quantitative real-time PCR and reverse-transcription PCR. Densitometry analyses showed that ATF6 expression was lower in Group 2H than that in Group 1 (*P* < .05), whereas the ATF6 levels in Groups 2R, 5, and 6 were significantly higher than those in Group 1. However, the ATF6 upregulation pattern was not seen with AICAR supplementation before H/R injury. The downstream genes of ER stress, such as VEGF, were positively correlated with ATF6 expression (Figure 3). The pattern of XBP-1 expression was similar to that of ATF6 expression. The different patterns of ATF6 and XBP-1 expression between metformin and AICAR supplementation suggest that they might act on different AMPK activation pathways. Although ATF6 was regulated by Grp78, activation of ATF6 could also initiate Grp78 gene transcription [23]. As shown in Figure 3, the expression pattern of the Grp78 transcript was similar to that of ATF6.

### 9.4. Effects of AMPK Activation on Bcl-2/Bax Ratio, Caspase-3, Cleaved PARP, and Apoptosis in HL-1 Cardiomyocytes by Western Blot Analysis and TUNEL Staining

**Figure 4:**
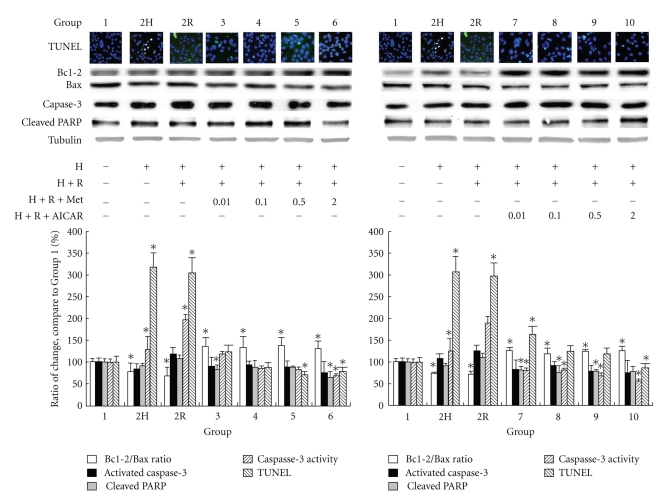
Effect of AMPK activation on cardiomyocytic apoptosis after cardioplegia-induced H/R injury. The percentage of apoptotic nuclei in different groups was determined by staining with terminal deoxynucleotidyl transferase-mediated dUTP-biotin nick end labeling (TUNEL). Protein levels of Bcl-2/Bax ratio, activated caspase-3, and cleaved poly-(ADP-ribose) polymerase were determined in the different groups by western blot analysis. Caspase-3 activity was also evaluated. (Results are representative of at least 3 separate experiments. *P < .05 compared with Group 1; H: hypoxia; R: reoxygenation; Met: metformin.)

Cardiomyocytic nuclei with nicked DNA were present in all groups (Figure 4). Apoptotic cell counts were expressed as a percentage of the total number of cardiomyocytic nuclei counted. The level of cardiomyocytic apoptosis (percent apoptotic nuclei) in Groups 2H and 2R was significantly higher than that in Group 1 (*P* < .05; Figure 4). This level decreased significantly with either AMPK activation in a dose-dependent manner (Groups 3–10 compared with Group 1). 

Besides GADD153, Bcl-2 family members also have an important role in modulation of ER stress-induced cell death [21], which possibly works through crosstalk between the ER and mitochondrial pathways for apoptosis. Figure 4 shows that the ratio of Bcl-2/Bax was significantly reduced in Groups 2R and 2H compared with that in Group 1 (*P* < .05). Furthermore, the reduced ratio was restored with either AMPK activator. The expression of activated caspase-3, caspase-3 activity, and cleaved PARP (a product of caspase-3) showed a similar pattern to that of the Bcl-2/Bax ratio.

## 10. Discussion

Metformin is a widely used antidiabetic drug. The UK Prospective Diabetes Study 34 revealed that the risk of diabetes-related death and the incidence of myocardial infarction associated with diabetes mellitus could be decreased with metformin therapy [24]. Sasaki et al. [25] also demonstrated that long-term administration of metformin had a direct cardioprotective effect on inhibition of cardiac remodeling and prevention of the progression of heart failure in canine model. Our results showed that metformin *per se *had antiapoptotic effect by reduction of endoplasmic reticulum stress on HL-1 cardiomyocytes during cardioplegia-induced H/R insult via activation of AMP kinase pathway.

In the myocardium, the ER participates in the maintenance of cellular calcium homeostasis and synthesis of proteins [15]. The ER is highly sensitive to alterations and perturbations in its environment; oxidative stress, accumulation of misfolded proteins and chemical toxins can all disrupt ER function resulting in ER stress. In order to survive the stress, specific signaling pathways are activated in the ER including the UPR, the ER-overload response, and the ER-associated degradation [26]. Thus, ER dysfunction might contribute to the pathogenesis of H/R injury. 

AMPK regulates cellular metabolism as well as response to a variety of signals not directly related to metabolism, such as ischemia, hypoxia, and oxidative stress [27]. AMPK also provided cardiac protection against tumor necrosis factor-alpha-triggered cardiomyocyte apoptosis through BCL-2 associated death promoter homolog protein and inflammation [28]. There are two known *α* subunit isoforms of AMPK; the *α*_1_ isoform appears to be expressed in all tissues, whereas the *α*_2_ isoform is expressed predominantly in skeletal and cardiac muscle [26]. The *α*_2_-containing AMPK complexes are the main contributors to AMPK activity in response to various types of AMPK stimulators [29]. However, the increase of AMPK protein levels dose not correlate with the increase of AMPK activity, because the increased protein is located in subcellular compartments that are unavailable for activation by AMPK kinase during stimulation [29]. In response to H/R stress, ER stress induced AMPK activation which regulated a variety of protein expression with the modulation of various eukaryotic initiation factors (eIFs) [30].

The initial UPR is characterized by the coordinated activation of multiple proteins to suppress protein synthesis, which will reduce the load of client proteins the ER must process. Eucaryotic translation eIF-2*α* kinase is one of the proteins that are activated in the initiation step, which is often referred to as the control point for protein synthesis [31]. When eIF-2*α* is phosphorylated, protein synthesis is inhibited. eIF-2*α* phosphorylation takes place during ischemia, apoptosis, viral infection, and after Ca^2+^ influx [31]. Therefore, eIF2*α* phosphorylation plays a significant role in the process of cell death after oxidative stress.

The other way to regulate UPR and ER stress is to control the transcription of UPR-responsive genes. ATF6 and XBP-1 can bind to the promoters of UPR-responsive genes under ER stress [32]. Transcription of UPR-responsive genes is induced when the cleaved form of ATF6 activates the XBP-1 promoter and excises an intron. Spliced XBP-1 then translocates into the nucleus, where it binds to its target sequence in the regulatory regions of the chaperone genes to induce their transcription. Therefore, signaling through ATF6 merges to induce XBP1 transcription and mRNA splicing [32]. 

Under nonstressed conditions, Grp78 functions as a master regulator of the UPR by binding to, and preventing, the activation of proximal stress sensors. When misfolded proteins accumulate in the cell, they bind to Grp78 and disrupt its interaction with the stress sensors, such as ATF6, resulting in their activation [21]. Two of the sensor systems that control the UPR are the IRE1/XBP-1 and ATF6 pathways. The XBP-1 pathway is required for efficient protein folding, maturation, and degradation in the ER and implies the existence of subsets of UPR target genes as defined by their dependence on XBP-1 [33]. Within 2 hours of hypoxic treatment in the present study, there was an initial drop of endogenous ATF6 protein level, in agreement with proteolytic cleavage, as reported previously [23]. At 16 hours of reoxygenation following hypoxic stress, and an additional, faster-migrating form was detected. With prolonged treatment the total amount of ATF6 also increased. Li et al. suggested that the faster-migrating form of ATF6 might be generated from preformed ATF6 through alternative splicing, since splicing of the short transmembrane domain from ATF6 could convert it from a predominantly membrane-associated form to a soluble form [23].

The UPR ensures the efficient translocation of newly synthesized peptides across the ER membrane and their subsequent folding, maturation, and transport by activating the expression of chaperone genes [32]. The second step of the UPR is to increase translocation and degradation of misfolded proteins in the ER by triggering the synthesis of ER chaperone proteins, such as Grp78, to rescue the cells [34]. During ER stress, there is increased expression of the protective intra-ER molecular chaperone, Grp78, which is intended to compensate for damage [35]. Otherwise, the cell dies by apoptosis. 

The ER is a repository for both pro- and antiapoptotic molecules. The known proapoptotic molecules include caspase-12 and GADD153 [21], whereas the antiapoptotic molecules identified to date include the ER chaperone proteins Grp78 and calreticulin [34]. ER stress induces the expression of GADD153, a transcription factor that participates in ER-mediated apoptosis [21], which promote ER stress-induced cell death by downregulating Bcl-2 expression [36].

In addition, a significant fraction of endogenous Bcl-2 family members including Bcl-2, Bcl-xL, and Bax has been shown to be associated with the ER, suggesting that Bcl-2 family proteins operate in the ER to regulate calcium homeostasis and apoptotic cell death [37]. Bcl-2 family proteins function in the maintenance of Ca^2+^ homeostasis and regulate the crosstalk between the ER and mitochondria. The balance between these various Bcl-2 family members acts as an “apoptotic rheostat” that modulates the transition of the cell from survival to death [21]. The limitation of our study was searching for possible antiapoptotic pathway and treatment during cardioplegia-induced cardiomyocytic H/R insult. However our results revealed that AMPK activation with metformin and AICAR could reduce cardiomyocytic apoptosis during cardioplegia-induced H/R insult. However, macroautophagy could also be stimulated with these antiapoptotic treatments [38], which had both beneficial and detrimental roles in the myocardium. The complex crosstalks between apoptosis and autophagy [39] are beyond the scope of our present study, which prompts further studies to clarify the “survival,” in stead of antiapoptotic, benefit of AMPK activation during cardioplegia-induced cardiomyocyte H/R insult.

## 11. Conclusions

Despite the limitation of using HL-1 cardiomyocytes in stead of primary cardiomyocytes in this study as model, the findings of this study demonstrate that metformin and AICAR decrease ER stress and increase antiapoptotic proteins and gene expression through AMPK. In addition, metformin and AICAR prevent caspase-12 and -3 activation and the occurrence of cardiomyocytic apoptosis after cardioplegia-induced H/R injury. Our findings suggest that blocking ER stress and UPR activation through AMPK could be useful in the design of new pharmacological approaches to prevent cardiomyocytic damage after cardioplegia-induced H/R insult.
